# Dysfunctional sleep-related cognition and anxiety mediate the relationship between multidimensional perfectionism and insomnia symptoms

**DOI:** 10.1007/s10339-019-00937-8

**Published:** 2019-10-26

**Authors:** Umair Akram, Maria Gardani, Dieter Riemann, Asha Akram, Sarah F. Allen, Lambros Lazuras, Anna F. Johann

**Affiliations:** 1 Department of Psychology, Sociology and Politics, Sheffield Hallam University, Collegiate Crescent, Sheffield, South Yorkshire S10 2BP UK; 2 Nuffield Department of Clinical Neurosciences, University of Oxford, Oxford, UK; 3 School of Psychology, University of Glasgow, Glasgow, UK; 4 Department of Psychiatry and Psychotherapy, Faculty of Medicine, Medical Center – University of Freiburg, University of Freiburg, Freiburg, Germany; 5 Department of Psychology, The University of Sheffield, Sheffield, UK; 6 Department of Health Sciences, University of York, York, UK; 7 Medical Psychology and Medical Sociology, Faculty of Medicine, University of Freiburg, Freiburg, Germany

**Keywords:** Personality, Perfectionism, Sleep, Insomnia, Dysfunctional cognition

## Abstract

Perfectionism is one of several personality traits associated with insomnia. Whilst research has examined the relationships between perfectionism and insomnia, the mediating role of dysfunctional sleep-related cognition (i.e. sleep-related worry and dysfunctional beliefs about the biological attribution of and consequences of poor sleep) has yet to be examined. This study aimed to determine whether aspects of multidimensional perfectionism were related to increased reporting of insomnia symptoms. In addition, the potential mediating role of dysfunctional sleep-related cognition and anxiety symptoms was examined. Members of the general population (*N* = 624) completed the Dysfunctional Beliefs and Attitudes About Sleep Scale, the Insomnia Severity Index, the Hospital Anxiety and Depression Scale, and the Multidimensional Perfectionism Scale. The results showed that perfectionism dimensions, anxiety symptoms, and dysfunctional sleep-related cognition were significantly associated with insomnia symptoms. Regression-based mediation analyses further showed that both dysfunctional sleep-related cognition and anxiety significantly mediated the associations between insomnia symptoms and three perfectionism dimensions (i.e. doubts about action, parental expectations, and parental criticism). The experience of perfectionistic tendencies, anxiety, and dysfunctional sleep-related cognition may initiate behavioural strategies (e.g. daytime napping) when faced with an acute sleep problem. However, these strategies may serve to transition insomnia from an acute to a chronic condition.

**Electronic supplementary material:**

The online version of this article (10.1007/s10339-019-00937-8) contains supplementary material, which is available to authorized users.

## Introduction

Insomnia is a prevalent sleep disorder affecting up to 10% of adults at disorder level and 30% at symptom level (Espie et al. 2012; Morin et al. 2006). Symptoms of insomnia including difficulty with sleep initiation, maintenance and/or early morning awakening, and significant impairment to daytime functioning often lead to impaired quality of life (Kyle et al. 2010). Various predisposing, precipitating, and perpetuating factors are proposed to influence the onset of insomnia which may be behavioural, biological, environmental, or psychological in nature (Spielman et al. 1987). Considering this, an individual’s personality may inherently act as a predisposing factor in relation to the onset of insomnia.

Perfectionism, defined as the tendency to set excessively high standards for oneself and to engage in overly critical self-evaluations (Frost et al. 1990), has been frequently associated with poor sleep and insomnia (Akram et al. 2015; Akram et al. 2017; Azevedo et al. 2010; Jansson-Fröjmark and Linton 2007; Johann et al. 2017; Lundh et al. 1994; Vincent and Walker 2000; Spiegelhalder et al. 2012). Specifically, relationships between insomnia and doubts about action, parental criticism, concern over mistakes, personal standards, and socially prescribed perfectionism have been evidenced. Moreover, recent research has evidenced that the relationship between perfectionism and insomnia may be mediated by emotional distress (Jansson-Fröjmark and Linton 2007), stress perception and emotion regulation(Brand et al. 2015), counterfactual thinking (Schmidt et al. 2018), and symptoms of anxiety, but not depression (Akram et al. 2015; Akram et al. 2017).

It has been theorized that perfectionistic (i.e. a disposition for excessive critical self-evaluation and high standards for oneself) individuals display a tendency to be overly concerned with the negative effects an acute sleep disturbance may have on their daytime performance (Lundh and Broman 2000). At night, this could transition into a negative thought cycle consisting of worry, rumination, and negative expectations/dysfunctional beliefs concerning sleep. In turn, this may eventually lead to sleep initiation and maintenance difficulty facilitating the transition from an acute to chronic sleep disturbance (Frost et al. 1990; Lundh et al. 1994).

Dysfunctional sleep-related cognitions include erroneous expectations about sleep requirements, exaggerated beliefs concerning the daytime consequences of disturbed sleep, worry and helplessness related to sleep, and faulty beliefs concerned with sleep medication and biological attribution of disturbed sleep (Morin et al. 2007). According to several aetiological models (e.g. 18), dysfunctional cognitions of this nature are instrumental in the development of a chronic sleep disturbance such as insomnia. This may arise through an interaction between negatively toned cognitive activity (e.g. worry and rumination) and autonomic hyperarousal which is mediated by emotional dysregulation in the form of extreme and arousing negative or positive emotions (Harvey 2002; Baglioni et al. 2010). Considering the nature of perfectionism in relation to sleep (i.e. an over-concern with sleep) and evidence of deficits in emotion regulation (Spiegelhalder et al. 2012), it is likely that those who exhibit perfectionist tendencies experience dysfunctional cognitions related to sleep. However, research has yet to explore this proposition.

This exploratory study aimed to investigate the relationship between facets of multidimensional perfectionism and insomnia, whilst incorporating the mediating role of dysfunctional sleep-related cognition and symptoms of anxiety. Specifically, we aimed to determine whether: (i) facets of multidimensional perfectionism were related to increased reporting of insomnia symptoms, and (ii) whether any empirically supported relationships remained after accounting for dysfunctional sleep-related cognitions and symptoms of anxiety. The present study is the first to examine the role of dysfunctional beliefs and attitudes about sleep in relation to perfectionism. Here, we expect dysfunctional beliefs about sleep and anxiety to mediate the relationship between facets of perfectionism and insomnia symptoms. However, considering mixed evidence concerning which facets of perfectionism are specifically related to insomnia, no a priori hypotheses are made in relation to this question.

## Materials and methods

### Sample and procedure

A cross-sectional online questionnaire-based study was implemented comprising questions designed to assess the relationship between facets of perfectionism, dysfunctional beliefs about sleep, and symptoms of anxiety and insomnia. The study was approved by the [Masked for review] University Research Ethics Committee, and all participants provided informed consent.

The survey was advertised to: (a) members of the general population through social media, “call for participants” (website) and (b) students at four Northern UK universities, through each institution’s course participation scheme. Seven hundred and thirty-two participants began the survey which was delivered using the Qualtrics platform (Qualtrics, Provo, UT), and 624 respondents provided complete data (mean age = 24.00 ± 10.85, range 18–76; 75% female; 86% White; 5% Chinese; 2% Pakistani; 2% Asian Other; 2% Indian; 1% African; 3% Other; 67% student) which was included into analysis. Students who requested course credit (46%) were remunerated on completion.

### Measures

The 16-item version of the Dysfunctional Beliefs and Attitudes About Sleep Scale (DBAS) (Morin et al. 2007) assessed sleep-related cognitions. The measure contains 16 items assessing beliefs related to consequences of poor sleep, beliefs related to worry/helplessness about insomnia and its effects, beliefs related to expectations about sleep, and beliefs about medication use. Items are scored on a scale ranging from 0 (strongly disagree) to 10 (strongly agree). The total score for the measure is calculated as the average item score, such that DBAS total scores range from 0 to 10. Higher scores indicate greater dysfunctional beliefs. Assessment of internal consistency yielded a Cronbach’s alpha of .89. Considering the asymmetry in the number of subscale items (ranging from 2 to 6) and the exploratory nature of the study, we chose to use the composite score.

The original version of the Frost Multidimensional Perfectionism Scale (F-MPS) (Frost et al. 1990) assessed different aspects of perfectionism. The 35-item F-MPS assesses six components on 5-point likert scales. Scores for each component range as follows: concern over mistakes (CM) 9–45; doubts about action (DA) 4–20; parental expectations (PE) 5–25; parental criticism (PC) 4–20; organization (ORG) 30; and personal standards (PS) 7–35. Higher scores represent a greater tendency towards perfectionism. Internal consistency assessment yielded a Cronbach’s *α* of .92 for the subscale CM; .82 for DA; .84 for PE; .83 for PC; .90 for ORG; and .83 for PS.

Insomnia symptoms were assessed using the Insomnia Severity Index (Bastien et al. 2001). The ISI consists of 7 items examining the severity of insomnia symptoms over the past 2 weeks including difficulty initiating and maintaining sleep and awakening too early. Items are scored on a 5-point Likert scale, with total scores ranging from 0 to 28. Higher scores represent greater insomnia severity. Assessment of internal consistency yielded a Cronbach’s alpha of .87.

Symptoms of anxiety were assessed using the anxiety subscale of the Hospital Anxiety and Depression Scale [HADS-A (Zigmond and Snaith 1983)], consisting of 7 items scored between 0 and 3, with a maximum score of 21. Higher scores represent greater anxiety. A score between 8–10 indicates mild; 11–14 moderate; and 15–21 severe symptom severity, whereas ≤ 7 indicates a normal (non-case) score. Assessment of internal consistency yielded a Cronbach’s alpha of .87.

### Statistical analyses

Bootstrapped hierarchical linear regression analysis was used to assess the association of perfectionism dimensions, dysfunctional sleep-related cognition, and symptoms of anxiety and insomnia. Bootstrapping is a robust alternative to standard parametric estimates, when the assumptions around the latter may be violated (Fox 2008). Finally, regression-based multiple mediation analysis (Hayes 2013; Preacher and Hayes 2008) was used to assess the mediating effect of dysfunctional sleep-related cognition and anxiety on the association between perfectionism dimensions and insomnia symptoms. All data were analysed in IBM SPSS v.24.0 (IBM Corp., Armonk, NT, USA). Significance was considered at the *p* < 0.05 level.

## Results

Mean scores DBAS, ISI, HADS-A, and F-MPS for the final sample are presented in Table 1.

**Table 1:** Means and standard deviations (SD) for DBAS, ISI, HADS-A, and F-MPS scores

	Mean (± SD)	Ranges
Insomnia Severity Index	5.33 ± 5.35	0–28
DBAS	5.51 ± 1.81	0–10
HADS-A	8.50 ± 4.62	0–21
*Perfectionism*		
Concern over mistakes	23.19 ± 8.28	9–45
Doubts about action	12.01 ± 3.83	4–20
Parental expectations	12.64 ± 4.55	5–25
Parental criticism	8.46 ± 3.78	4–20
Organization	21.52 ± 5.03	6–30
Personal standards	18.33 ± 4.85	6–30

*DBAS* Dysfunctional Beliefs and Attitudes About Sleep Scale, *ISI* Insomnia Severity Index, *HADS*-*A* Anxiety, *F*-*MPS* Multidimensional Perfectionism Scale

### Direct effects of perfectionism dimensions, dysfunctional sleep-related cognition, and anxiety on insomnia symptoms

A bootstrapped hierarchical linear regression analysis was used to assess the associations between perfectionism dimensions, dysfunctional sleep-related cognition, anxiety and self-reported insomnia symptoms. Bootstrapping with 1000 bias-corrected and accelerated (BCa) resamples (Mallinckrodt et al. 2006) and 95% confidence intervals was used, as this analytic approach allows for a more robust estimation of the regression coefficients (Randles et al. 2010). The overall model was statistically significant (*F* = 44.00, *p* < .001) and predicted 40.8% of the variance (adjusted *R*^2^) of insomnia symptoms—a large multivariate effect size (*f*^2^ = 0.68) (Cohen 2013). Tolerance levels (> .334) were above the recommended cut-off points for multicollinearity, thus showing that the predictor variables were independent of each other. The first step of this analysis included demographic characteristics (age and sex) and the six F-MPS dimensions. At this step, the model predicted 15.3% of the variance of insomnia symptoms and the significant predictors included doubts about actions (*β* = .273, *p* < .001), organization (*β* = − .137, *p* = .001), and parental expectations (*β* = − .116, *p* < .05) and criticism (*β* = .196, *p* = .001). At the second step, we added dysfunctional sleep-related cognition, which had a significant effect (*β* = .495, *p* < .001) and increased predicted variance by 19.9% (*F*_change_ = 191.99, *p* < .001). The standardized beta coefficients of the four perfectionism dimensions that were significant in the first step of the analysis were retained due to their statistical significance. Finally, anxiety symptom scores were added in the third step of the analysis and further increased predicted variance by 5.5% (*F*_change_ = 57.64, *p* < .001). At this step, the variables that were significantly associated with increased insomnia symptoms included lower scores in organization (*β* = − .072, *p* < .05) and concern over mistakes (*β* = − .136, *p* < .05), and higher scores in parental criticism (*β* = .178, *p* < .001), dysfunctional sleep-related cognition (*β* = .426, *p* < .001), and anxiety (*β* = .312, *p* < .001). The results are summarized in Table 2.

**Table 2:** Direct effects of perfectionism dimensions, dysfunctional sleep-related cognition, and anxiety on insomnia symptoms

Predictor	*B*	*β*	BCa 95% CIs for *B*	Adjusted *R*^2^ (%)
*Step 1*				15.3
Age	.008	.016	− .025, .043	
Sex	.671	.054	− .379, 1.633	
Doubts about actions	.382	.273***	.235, .502	
Organization	− .145	− .137**	− .245, − .054	
Concern over mistakes	.013	.020	− .081, .111	
Parental expectations	− .136	− .116*	− .264, − .027	
Parental criticism	.277	.196**	.108, .475	
Personal standards	.072	.065	− .047, .190	
*Step 2*				35.4
Age	.024	.048	− .006, .056	
Sex	− .072	− .006	− .959, .741	
Doubts about actions	.219	.157***	.080, .342	
Organization	− .088	− .083*	− .184, .003	
Concern over mistakes	− .042	− .065	− .124, .038	
Parental expectations	− .121	− .103*	− .224, − .029	
Parental criticism	.248	.175**	.117, .398	
Personal standards	.061	.056	− .051, .175	
DBAS	1.460	.495***	1.225, 1.703	
*Step 3*				40.8
Age	.027	.056	− 5.595, .056	
Sex	− .335	− .027	− 1.151, .454	
Doubts about actions	.046	.033	− .078, .164,	
Organization	− .077	− .072*	− .169, .011	
Concern over mistakes	− .088	− .136*	− .164, − .006	
Parental expectations	− .093	− .079	− .192, − .004	
Parental criticism	.252	.178***	.135, .389	
Personal standards	.069	.063	− .033, .174	
DBAS	1.258	.426***	.992, 1.528	
HADS-A	.362	.312***	.258, .467	

*DBAS* Dysfunctional Beliefs and Attitudes About Sleep Scale, *HADS*-*A* Anxiety, *F*-*MPS* Multidimensional Perfectionism Scale

*p < .05; **p < .005; ***p < .001

### Indirect effects of perfectionism dimensions on insomnia symptoms

**Fig. 1:**
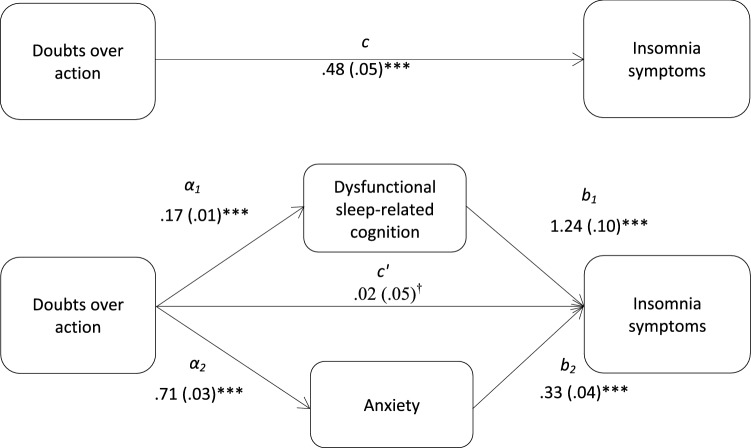
Total and indirect effect of doubts over action on insomnia symptoms. Total (*c*) and indirect effect (*c*′) of doubts over action on insomnia symptoms are shown; unstandardized path coefficients are presented, with standard errors in brackets; ^†^the direct effect (*c*′) of doubts over action on insomnia symptoms was *n*–*s*; *p < .05; **p < .005; ***p < .001. The total variance (adjusted *R*^2^) predicted by the model was 38.3% (*p* < .001)

**Fig. 2:**
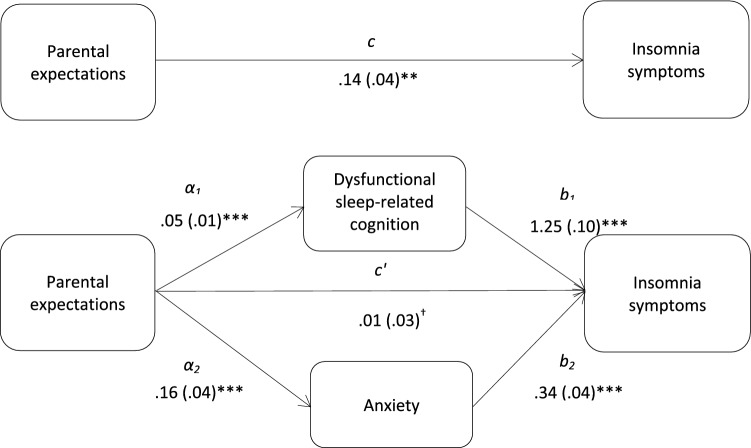
Total and indirect effect of parental expectations on insomnia symptoms. Total (*c*) and indirect effect (*c*′) of parental expectations on insomnia symptoms are shown; unstandardized path coefficients are presented, with standard errors in brackets; ^†^the direct effect (*c’*) of parental expectations on insomnia symptoms was n-s; *p < .05; **p < .005; ***p < .001. The total variance (adjusted *R*^2^) predicted by the model was 38.3% (*p* < .001)

**Fig. 3:**
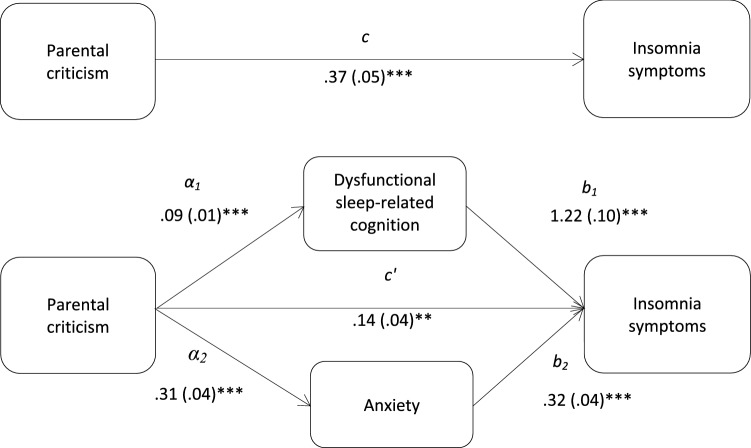
Total (*c*) and the indirect effect (*c*′) of parental criticism on insomnia symptoms are shown; unstandardized path coefficients are presented, with standard errors in brackets; *p < .05; **p < .005; ***p < .001. The total variance (adjusted *R*^2^) predicted by the model was 39.3% (*p* < .001)

Hayes (2013) multiple mediation method was used to determine the indirect effects of perfectionism dimension on insomnia symptoms, via dysfunctional sleep-related cognition and anxiety. Bootstrapping with 1000 bias-corrected and accelerate resamples and 95% confidence intervals were used, and the Sobel test (*z*) was used to indicate the hypothesized mediation effects. Four multiple mediation models were tested examining the mediation effects of dysfunctional sleep-related cognition and anxiety on the associations between doubts about actions and insomnia symptoms (Fig. 1); parental expectations and insomnia symptoms (Fig. 2); parental criticism and insomnia symptoms (Fig. 3); and organization and insomnia symptoms.

The results demonstrated that dysfunctional sleep-related cognition and anxiety significantly mediated the relationships between insomnia symptoms and doubts about action (*z*_DBAS_ = 7.67, *p* < .001; *z*_anxiety_ = 6.67, *p* < .001), parental expectations (*z*_DBAS_ = 3.55, *p* < .001; *z*_anxiety_ = 3.66, *p* < .001), and parental criticism (*z*_DBAS_ = 4.82, *p* < .001; *z*_anxiety_ = 5.10, *p* < .001). Further analyses showed that the mediation effects of dysfunctional sleep-related cognition and anxiety did not differ significantly. Dysfunctional sleep-related cognition did not mediate the association between organization and insomnia symptoms. The results from the multiple mediation analysis are presented in Table 3.

**Table 3:** Indirect effects of perfectionism dimensions on insomnia symptoms, via dysfunctional sleep-related cognition and anxiety

	Product of coefficient			BCa 95% CIs for *B*	
Mediator	Parameter estimate (*B*)	SE	*Z*	Lower	Upper
*Doubts about actions and insomnia symptoms*					
DBAS	0.219	0.028	7.67***	0.159	0.288
HADS-A	0.242	0.036	6.67***	0.164	0.334
*Parental expectations and insomnia symptoms*					
DBAS	0.073	0.020	3.55***	0.035	0.122
HADS-A	0.056	0.015	3.66***	0.029	0.102
*Parental criticism and insomnia symptoms*					
DBAS	0.121	0.025	4.82***	0.073	0.184
HADS-A	0.102	0.020	5.10***	0.058	0.156

*DBAS* Dysfunctional Beliefs and Attitudes About Sleep Scale, *ISI* Insomnia Severity Index, *HADS*-*A* Anxiety

*p < .05; **p < .005; ***p < .001

## Discussion

The present study examined the relationship between facets of multidimensional perfectionism and insomnia, and whether this relationship is mediated by symptoms of anxiety and higher scores in dysfunctional sleep-related cognition. The results showed that individuals presenting symptoms of insomnia tended to also report a greater degree of doubts about action (e.g. “I usually have doubts about the simple everyday things that I do”), parental criticism (e.g. “My parents never tried to understand my mistakes”) and parental expectations (e.g. “My parents want me to be the best at everything”) and reduced organization (e.g. “I try to be an organized person”). Our results are in line with the existing literature evidencing significant relationships between multidimensional perfectionism and symptoms of insomnia (Akram et al. 2015, 2017; Azevedo et al. 2010; Jansson-Fröjmark and Linton 2007; Johann et al. 2017; Lundh et al. 1994; Vincent and Walker 2000). Regression-based multiple mediation analyses further showed that, with the exception of organization, the association between perfectionism dimensions and insomnia symptoms in the present study was significantly mediated by higher symptoms of anxiety and dysfunctional beliefs and attitudes about sleep. Whilst anxiety is known to mediate the perfectionism–insomnia relationship(Akram et al. 2015, 2017; Jansson-Fröjmark and Linton 2007), this is the first study to highlight the mediating role of dysfunctional sleep cognition between perfectionism dimensions and insomnia symptoms.

Individuals who excessively doubt their own actions and worry about parental criticism have been theorized to experience heightened arousal during the pre-sleep period, which contributes to delayed sleep onset (Lundh et al. 1994; Vincent and Walker 2000). This may result from worry and rumination commonly associated with higher perfectionistic thinking (Randles et al. 2010). Indeed, in the occurrence of an acute bout of poor sleep, individuals who are high on these facets of perfectionism may spend a disproportionate amount of time critically evaluating their sleep and daytime performance (Akram et al. 2015). Here, excessive worry and exaggerated notions about the effects of transient sleep loss, daytime consequences of poor sleep, and the biological attribution of these problems are likely to influence the evaluation of sleep in a negative manner. In turn, the perception that poor sleep hinders daytime functioning could lead to doubts about actions which are to be performed during the day. In addition, anxious symptoms may serve to exacerbate pre-existing worry and ruminative thinking specific to sleep amongst individuals with insomnia. Eventually, a negative thought cycle is theorized to occur, where doubts about actions and performance during the day transition into the night consequently fuelling increased pre-sleep arousal and delayed sleep onset (Schmidt et al. 2018). As a result, those high in both perfectionistic tendencies and dysfunctional sleep-related cognition may alter behavioural (i.e. increased time in bed through napping or attempting sleep earlier than normal) strategies to compensate for their sleep deficit and consequently aim to *perfect* sleep. Here, such individuals may actively attempt to force the initiation of sleep, a tactic known as sleep effort (Baglioni et al. 2010). However, in reality, engaging in sleep effort serves only to transition insomnia from an acute to a chronic problem (Baglioni et al. 2010).

As recently highlighted (Johann et al. 2018), perfectionism may increase the risk of dropout from cognitive behavioural therapy for insomnia (CBT-I). With that in mind, those who present with perfectionistic tendencies may benefit from a modified version of CBT-I and/or CBT for perfectionism (Johann et al. 2018; Akram 2018), one which perhaps places greater emphasis on correcting dysfunctional beliefs about sleep and alleviating perfectionistic thoughts concerning sleep and symptoms of anxiety. By providing perfectionists with the correct information about sleep, we may prevent acute sleep difficulty from transitioning into a long-term problem by preventing dysfunctional cognitions which facilitate increased behavioural efforts to sleep. Indeed, as evidenced recently, this may be achieved through a single session of CBT-I supplemented with a self-help guide (Ellis et al. 2015).

Several limitations of the current study should be noted. First, the present sample was mostly female and predominantly students, and therefore, the outcomes may not be entirely generalizable to males and the general population. Whilst a more representative sample should be used in further research, it is relevant to note that women are more likely to experience insomnia compared to men (Zhang and Wing 2006), whereas sex differences in relation to perfection appear to be mixed (Hewitt et al. 1991). Next, considering the cross-sectional nature of the current data, the results remain vulnerable to an inflation bias between variables (Parker et al. 2001). Moreover, cross-sectional data limit causally and directionally in determining whether perfectionistic tendencies predict dysfunctional beliefs and attitudes about sleep. Additionally, we did not control for symptoms of other sleep disorders other than insomnia (e.g. apnoea, narcolepsy) or psychiatric symptoms (e.g. anxiety, depression) which may have influenced the outcomes. Finally, the current data are limited to a subjective measure of sleep. With that in mind, objectively defined short sleep duration and behavioural traits have been associated with obsessive punctuality amongst sleep disorder patients (Spiegelhalder et al. 2012). Therefore, it would be beneficial to re-examine the current research questions in conjunction with an objective sleep assessment (i.e. actigraphy) and subjective sleep log.

In summary, the present study demonstrated that the relationship between multidimensional perfectionism and insomnia symptoms was mediated by dysfunctional sleep-related cognition and symptoms of anxiety. Moving forward, to confirm whether individuals high in perfectionism and dysfunctional beliefs about sleep alter behavioural efforts to sleep, future research may wish to administer the Glasgow Sleep Effort Scale (Broomfield and Espie 2005) when further examining the current research questions. Finally, given the role of perfectionism in relation to sleep, future research should investigate the potential of modified CBT for insomnia and perfectionism (respectively), with the goal of alleviating improving sleep and alleviating dysfunctional sleep-related cognitions (Johann et al. 2018).

## Electronic supplementary material

Below is the link to the electronic supplementary material.

Supplementary material 1 (DOCX 13 kb)
