# Supplementary material for: Dysfunctional sleep-related cognition and anxiety mediate the relationship between multidimensional perfectionism and insomnia symptoms
Source: Cogn Process. 2019 Oct 26;21(1):141–8. doi: 10.1007/s10339-019-00937-8 (PMC7002331; doi:10.1007/s10339-019-00937-8)
Supplement: Supplementary file 1 — Supplementary material 1 (DOCX 13 kb) [file 10339_2019_937_MOESM1_ESM.docx]

**Supplementary material: Dysfunctional sleep-related cognition and anxiety mediate the relationship between multidimensional perfectionism and insomnia symptoms.**

**Sup1.** Table displaying DBAS subscale means and standard deviations for the final sample.

| **Supplementary Table 1** | | |
| --- | --- | --- |
| Means and standard deviations (SD) for DBAS, ISI, and F-MPS scores. | | |
|  | Mean (±SD) | Ranges |
|  |  |  |
| **Dysfunctional Beliefs and Attitudes About Sleep:** |  |  |
| Composite Score | 5.51 ± 1.81 | 0-10 |
| Worry and Helplessness | 5.05 ± 2.35 | 0-10 |
| Consequences of Poor Sleep | 6.00 ± 2.11 | 0-10 |
| Medication Use | 4.28 ± 2.28 | 0-10 |
| Expectations About Sleep | 7.52 ± 2.54 | 0-10 |
|  |  |  |
| Note: DBAS, Dysfunctional Beliefs and Attitudes about Sleep Scale. The 16-item version of the Dysfunctional Beliefs and Attitudes About Sleep Scale (DBAS: Morin et al., 2007) assessed sleep-related cognitions. The measure contains four subscales with: five items assessing beliefs related to consequences of poor sleep (e.g. *“cannot function without a good night”, “cancel obligations”*; six items assessing beliefs related to worry/helplessness about insomnia and its effects (e.g. *“worried about losing control of sleep”, “insomnia destroying life”*); two items assessing beliefs related to expectations about sleep (e.g. *“need eight hours of sleep”, “need to catch up on sleep loss”*); and three items assessing beliefs about medication use (e.g. *“better taking sleeping pills”, “insomnia resulting from chemical imbalance”*). Items are scored on a scale ranging from 0 (strongly disagree) to 10 (strongly agree). The composite score for the measure is calculated as the average item score from all subscales, such that DBAS total scores range from 0 to 10. Likewise, subscales are calculated in the same manner. Higher scores indicate greater dysfunctional beliefs. Assessment of internal consistency yielded a Cronbach’s alpha of .89 for the composite score, and: .85 for the worry/helplessness; .81 for the consequences; .68 for the medication use; and .64 for the expectation subscales. | | |

**Sup2.** Table displaying correlations between measures of dysfunctional beliefs about sleep, insomnia symptoms and multidimensional perfectionism.

| **Supplementary** **Table 2**  Correlations between measures of dysfunctional beliefs about sleep, insomnia symptoms and multidimensional perfectionism for all participants. | | | | | | | | | | | |
| --- | --- | --- | --- | --- | --- | --- | --- | --- | --- | --- | --- |
|  | 1. | 2. | 3. | 4. | 5. | 6. | 7. | 8. | 9. | 10. | 11. |
| 1. ISI |  |  |  |  |  |  |  |  |  |  |  |
| 2. DBAS Composite | .56** |  |  |  |  |  |  |  |  |  |  |
| 3. Worry/Helplessness | .67** | .89** |  |  |  |  |  |  |  |  |  |
| 4. Consequences | .36** | .87** | .63** |  |  |  |  |  |  |  |  |
| 5. Medication | .46** | .75** | .64** | .51** |  |  |  |  |  |  |  |
| 6. Expectations | -.03 | .41** | .10* | .45** | .10* |  |  |  |  |  |  |
| 7. CM | .28** | .35** | .38** | .25** | .29** | .03 |  |  |  |  |  |
| 8. DA | .35** | .37** | .41** | .28** | .27** | .02 | .64** |  |  |  |  |
| 9. PE | .12* | .15** | .16** | .10* | .16** | -.03 | .48** | .30** |  |  |  |
| 10. PC | .26** | .21** | .28** | .12* | .20** | -.10* | .54** | .42** | .69** |  |  |
| 11. ORG | -.11* | -.07 | -.10* | -.03 | -.10* | .08* | .07 | .05 | .07 | -.04 |  |
| 12. PS | .15** | .17** | .21** | .12* | -.14** | -.06 | .61** | .36** | .38** | .32** | .31** |
| *Note:* DBAS: Dysfunctional Beliefs About Sleep Scale; ISI: Insomnia Severity Index; CM, Concern Over Mistakes; D, Doubts About Action; PE, Parental Expectation; PC, Parental Criticism; ORG, Organisation; PS, Personal Standards.  * Sig at < .05, ** Sig at < .001 | | | | | | | | | | | |

**Sup3.** Table displaying linear regression analyses, with each DBAS subscale as dependant variables, to determine the predictive value of perfectionism facets which were associated with DBAS scores after controlling for insomnia symptoms.

| **Supplementary** **Table 3**  Linear regression analyses with individual dysfunctional beliefs about sleep subscales as the dependent variables; multidimensional perfectionism and insomnia symptoms as predictors | | | | |
| --- | --- | --- | --- | --- |
| *Predictors* | R² | β | *t* | Sig. |
| **[A] Worry and helplessness** |  |  |  |  |
| Step 1 | .21 |  |  |  |
| Concern over mistakes |  | .16 | 2.73 | .007** |
| Doubts about action |  | .28 | 5.83 | .001*** |
| Parental expectation |  | -.09 | -1.68 | .094 |
| Parental criticism |  | .11 | 2.11 | .035* |
| Personal standards |  | .04 | 0.90 | .37 |
| Organization |  | -.13 | -3.27 | .001*** |
| Step 2 | .50 |  |  |  |
| Concern over mistakes |  | .14 | 3.01 | .003** |
| Doubts about action |  | .12 | 3.06 | .002** |
| Parental expectation |  | -.02 | -0.40 | .69 |
| Parental criticism |  | .00 | 0.02 | .98 |
| Personal standards |  | .01 | 0.31 | .76 |
| Organization |  | -.05 | -1.70 | .09 |
| Insomnia symptoms |  | .58 | 18.65 | .001*** |
| **[B] Consequences of poor sleep** |  |  |  |  |
| Step 1 | .09 |  |  |  |
| Concern over mistakes |  | .16 | 2.58 | .01** |
| Doubts about action |  | .21 | 4.21 | .001*** |
| Parental expectation |  | .02 | 0.41 | .68 |
| Parental criticism |  | -.06 | -1.09 | .27 |
| Personal standards |  | -.05 | -0.97 | .33 |
| Step 2 | .17 |  |  |  |
| Concern over mistakes |  | .15 | 2.42 | .02* |
| Doubts about action |  | .13 | 2.69 | .007 |
| Parental expectation |  | .06 | 1.18 | .24 |
| Parental criticism |  | -.13 | -2.29 | .02* |
| Personal standards |  | -.05 | -1.02 | .31 |
| Insomnia symptoms |  | .30 | 7.61 | .001*** |
| **[C] Medication use** |  |  |  |  |
| Step 1 | .11 |  |  |  |
| Concern over mistakes |  | .19 | 2.99 | .003** |
| Doubts about action |  | .14 | 2.86 | .004** |
| Parental expectation |  | .01 | 0.23 | .82 |
| Parental criticism |  | .03 | 0.52 | .60 |
| Personal standards |  | -.01 | -.10 | .92 |
| Organization |  | -.12 | -2.99 | .003** |
| Step 2 | .25 |  |  |  |
| Concern over mistakes |  | .18 | 3.02 | .003** |
| Doubts about action |  | .04 | 0.74 | .46 |
| Parental expectation |  | .06 | 1.20 | .23 |
| Parental criticism |  | -.05 | -0.90 | .37 |
| Personal standards |  | -.03 | -0.56 | .58 |
| Organization |  | -.07 | -1.89 | .06 |
| Insomnia symptoms |  | .40 | 10.52 | .001*** |
| **[D] Expectations about sleep** |  |  |  |  |
| Step 1 | .02 |  |  |  |
| Parental criticism |  | -.10 | -2.49 | .053 |
| Organization |  | .08 | 1.94 | .013* |
| Step 2 | .02 |  |  |  |
| Parental criticism |  | -.10 | -2.41 | .054 |
| Organization |  | .08 | 1.93 | .02* |
| Insomnia symptoms |  | .00 | 0.10 | .99 |
| *Note:* * Sig at < .05, ** Sig at < .01, *** Sig at < .001 | | | | |
